# Research Progress on NK Cell Receptors and Their Signaling Pathways

**DOI:** 10.1155/2020/6437057

**Published:** 2020-07-24

**Authors:** Yingying Chen, Dan Lu, Alexey Churov, Rong Fu

**Affiliations:** ^1^Department of Hematology, General Hospital of Tianjin Medical University, China; ^2^Institute of Biology, Karelian Research Centre, Russian Academy of Sciences, Petrozavodsk, Russia

## Abstract

Natural killer cells (NK cells) play an important role in innate immunity. NK cells recognize self and nonself depending on the balance of activating receptors and inhibitory receptors. After binding to their ligands, NK cell receptors trigger subsequent signaling conduction and then determine whether NK is activated or inhibited. Furthermore, NK cell response includes cytotoxicity and cytokine release, which is tightly related to the activation of NK cell-activating receptors and the inhibition of inhibitory receptors on the surfaces of NK cells. The expression and function of NK cell surface receptors also alter in virus infection, tumor, and autoimmune diseases and influence the occurrence and development of diseases. So, it is important to understand the mechanism of recognition between NK receptors and their ligands in pathological conditions and the signaling pathways of NK cell receptors. This review mainly summarizes the research progress on NK cell surface receptors and their signal pathways.

## 1. Introduction

NK cells are crucial immune cells and enormously contribute to the innate immunity. NK cells can differentiate self from nonself by activating receptors and inhibitory receptors. NK cells exhibit natural cell cytotoxicity and directly destroy tumor cells or virally infected cells. Besides, NK cells play crucial roles in regulating various hematopoietic, inflammatory, and immune responses by secreting cytokines and chemokines [1, 2]. Therefore, it is necessary to understand the function of different surface NK cell receptors and their mechanisms of action. This article will summarize the existing research on NK cell receptors as well as their signaling pathways.

## 2. The Classification of NK Cell Receptors

Dozens of NK cell receptors have been discovered to date. These can be classified into the immunoglobulin superfamily (Ig-SF) and C-type lectin superfamily (CL-SF) according to their structure [3]. The Ig-SF includes killer cell immunoglobulin receptors (KIRs) [3, 4], leucocyte immunoglobulin-like receptors (LILRs/LIRs) [5], and natural cytotoxic receptors (NCRs) [6]. The CL-SF mainly includes killer cell lectin-like receptors (KLRs) [7].

NK cell receptors can be divided into two types according to functional classification [8]: inhibitory receptors and activating receptors. Inhibitory receptors mainly include KIR-2DL, KIR-3DL, CD94/NKG2A, and TIGIT. Activating receptors mainly contain KIR-2DS, KIR-3DS, NCR (NKp46, NKp44, and NKp30), NKG2D, 2B4, CD226, CD94/NKG2C, etc. In this volume, we will discuss NK cell receptors, respectively.

## 3. Inhibitory Receptors

NK cells express various inhibitory receptors. Most of inhibitory receptors, by identifying MHC class I molecules, conduct inhibitory signals to suppress NK cell function and participate in autoimmune tolerance under physiological conditions to avoid killing normal cells. In addition, some non-MHC-restricted inhibitory receptors are also focused on the immune escape of tumor cells and virally infected cells under pathological conditions.

### 3.1. Inhibitory Killer Cell Immunoglobulin Receptors (IKIRs)

KIRs belong to the Ig-SF. According to the structure of extracellular region, KIRs are divided into two categories, namely, KIR2D with two Ig-like domains and KIR3D with three Ig-like domains. KIR2DL and KIR3DL are inhibitory receptors that have longer intracellular tails with the immunoreceptor tyrosine-based inhibitory motifs (ITIMs) [4]. Other members are defined as an S to reflect their short ITIM-lacking intracellular region (KIR2DS and KIR3DS), which associate with adaptor proteins through the transmembrane region. These adaptor proteins help to deliver activating signals by means of immunoreceptor tyrosine-based activating motifs (ITAMs) in their intracellular region [9, 10]. The majorities of KIRs are highly specific for classic MHC-I molecules (HLA-A, HLA-B, and HLA-C) [4]. For instance, KIR2DL1, KIR2DL2, and KIR2DL3 are specific receptors of HLA-C molecules, and KIR3DL1 and KIR3DL2 can combine with HLA-A or HLA-B. Unlike other KIRs, KIR2DL4 recognizes both soluble and membrane HLA-G. However, in endosomes, only when KIR2DL4 binds to soluble HLA-G can the signals be transmitted [11].

**Figure 1:**
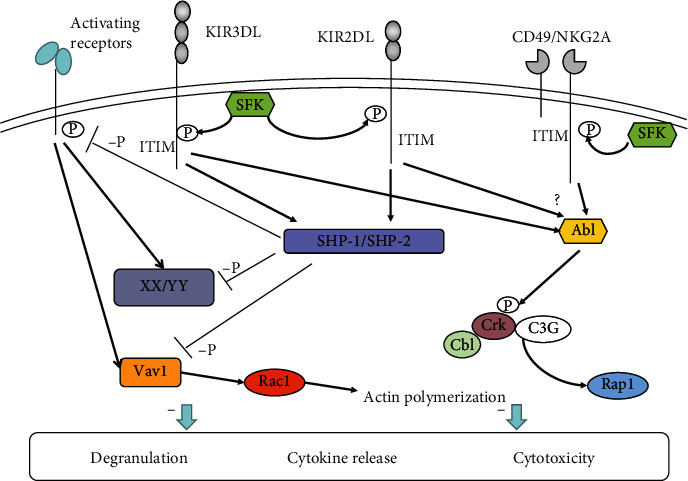
Signaling pathways of inhibitory receptors in NK cells. After KIR2DL, KIR3DL, and CD49/NKG2A bind to their corresponding ligands, the ITIM sequences are phosphorylated by Src-family kinase (SFK). Furthermore, the phosphorylated ITIM recruits SHP-1/SHP-2, which downregulates the phosphorylation level of the downstream signaling molecules (XX/YY) of activating receptors, including Vav1, thereby inhibiting NK cell function. In addition, Crk binds to Abl and is phosphorylated by Abl, which separates Crk from the Cbl-Crk-C3G complex, further inhibiting NK cell function.

When the inhibitory receptor recognizes its corresponding ligand, Src-family kinase (SFK) mediates the phosphorylation of ITIM sequences in the inhibitory receptor immediately [12]. After phosphorylation, ITIMs activate protein tyrosine phosphatases (PT-Pases), mainly including Src homology region 2-containing protein tyrosine phosphatase-1 (SHP-1) and Src homology region 2-containing protein tyrosine phosphatase-2 (SHP-2) [13–15]. As an effector molecule of inhibitory receptor, SHP-1 downregulates multiple activating signal molecules by dephosphorylation [16, 17] (Figure 1). Thus, SHP-1 plays a crucial role in initiating inhibitory signals and blocking activating signals, and the substrates of SHP-1 need to be further identified. During the repression of NK cells by ITIM-containing receptors, the tyrosine phosphorylation level of multiple proteins is downregulated [17]. Previously, it was viewed that the directly identified substrate of SHP-1 is Vav1. Vav1 can promote rac1-dependent cytoskeletal rearrangement, synapse formation, and receptor aggregation. However, SHP-1-catalyzed dephosphorylation of Vav1 does not depend on actin polymerization in inhibitory signaling [18]. This may suggest that ITIM-containing inhibitory receptors' repression of NK cell activation before the actin-dependent signals occurs and even before tyrosine phosphorylation of activating receptors [8, 18]. In 2016, researchers found that LAT and PLC*γ*1/2 served as the substrates of SHP-1 when NK cells were inhibited by MHC-restricted inhibitory receptor KILR2DL1 [19]. Besides, LAT could be ubiquitylated by c-TCbl and Cbl-b and degraded in inhibitory condition of NK cells [19]. They also confirmed that LAT:PLC*γ*1/2 complexes were necessary for degranulation and cytotoxicity of NK cells [19]. ITIM-containing receptors of NK cells are also involved in the downstream pathways when transmitting inhibitory signals. Peterson and Long found that when NK cells interacted with target cells expressing the MHC-I molecules, the adaptor Crk could associate with tyrosine kinase Abl and be phosphorylated [20]. Crk binding to Abl and phosphorylation are essential steps for the separation of Crk from the Cbl-Crk-C3G complex, a part of the activating signal pathway. These results point out that phosphorylation also plays a significant role in NK cell inhibitory signaling pathways (Figure 1). Overall, NK cell inhibitory receptors induce Vav1, LAT, and PLC*γ*1/2 dephosphorylation and Crk phosphorylation, which inhibit the NK cell activation signal and eventually inhibit NK cell activity. Moreover, inhibitory receptors, which recognize the MHC-I molecules, can provide a license for NK cell responsiveness [21]. The functions of inhibitory receptors, whatever inhibition or licensing, require the participation of activating receptor signal molecules. Therefore, the downstream molecules of SHP-1 and SHP-2 need to be observed further, and much work remains to be done in how inhibitory receptors conduct inhibitory signals and inhibit NK cell function. The connection between inhibitory receptors and activating receptors remains to be investigated further.

KIR2DL4 only carries a single ITIM in the intracellular tail, and its transmembrane region contains an arginine residue, which suggests that KIR2DL4 has dual functions of inhibition and activation [22]. Although functional KIR2DL4 is mainly located in endosomes, studies have demonstrated that IL-2 can transiently upregulate the expression levels of KIR2DL4 on NK cell surfaces, in vitro, and it is closely related to the NK cell function [23]. Further research showed that KIR2DL4 on the NK cell surface noncovalently associated with Fc*ε*RI*γ* via arginine residues and then activated ITAM signal transduction [24]. KIR2DL4 is expressed in endosomes and is associated with proinflammatory and angiogenic functions through the DNA-PKCs-Akt-NF-*κ*B signaling pathway [11, 25]. Studies found that KIR2DL4 can connect to SHP-1 and SHP-2 through pull-down experiments, suggesting that KIR2DL4 has inhibitory potential [22]. Moreover, other studies showed that the ITIMs of KIR2DL4 could inhibit the cytotoxic effect of NK cells, while functionally mutated SHP-1 did not block the cytotoxic effect of KIR2DL4 completely [15], suggesting that the phosphorylated ITIM of KIR2DL4 recruits SHP-2 instead of SHP-1. Further research showed that the tyrosine residue of the mutated ITIM motif did not thoroughly abrogate the function of KIR2DL4, which may be related to SHP-2 binding to the mutated ITIMs and nonphosphorylated ITIMs of KIR2DL4 on NK cells [15]. These results suggest that KIR inhibitory signal transduction is partially independent of SHP-1 or phosphotyrosine. Similarly, KIR2DL5 has typical ITIM sequences and an atypical ITIM in the intracellular region and can recruit both SHP-1 and SHP-2 simultaneously [26]. The cytotoxicity of NK cells can be suppressed by functionally mutated SHP-2 instead of functionally mutated SHP-1. These studies indicate that KIR2DL5 might have a more obvious inhibitory function [15]. In addition, KIR3DL1 directly binds to SHP-2 through conformational changes in the intracellular region, inhibiting target cell conjugation and cytotoxicity function [14, 27].

### 3.2. CD94/NKG2A and LIRs

Killer cell lectin-like receptor (KLR, CD49/NKG2) is a heterodimeric receptor that combines CD94 with different NKG2 family members through disulfide bonds [28]. KLR can be detected on most NK cell membranes. The NKG2A intracellular segment contains an ITIM, which transduces inhibitory signals [7]. The NKG2C intracellular segment is shorter and does not contain ITIM sequences, but it can bind to the ITAM-containing adaptor proteins to conduct activation signals [29]. The ligands of both CD49/NKG2A and CD49/NKG2C are types of the nonclassical MHC molecule, HLA-E [30]. Under normal conditions, HLA-E has a greater affinity for NKG2A than for NKG2C. Under stress, the HLA-E molecules of “stressed” cells bind to the polypeptide containing heat shock protein 60 (HSP60), which decreases HLA-E affinity with NKG2A and increases affinity with NKG2C, thus activating NK cells [31]. Other studies have shown that NKG2A is associated with immune escape. Senescent dermal fibroblasts express HLA-E, which binds to the NKG2A and suppresses the immune reaction of senescent cells [32]. CD94/NKG2A could inhibit the synergistic effect of activating receptors NKG2D and 2B4 [33, 34]. Moreover, CD94/NKG2A complex could inhibit the CD16-dependent activation of Syk and ERK [35]. Those results showed that the activation signals of NK cells can be blocked by inhibitory receptors in multiple levels.

LIRs belong to the Ig-SF, which have ITIM sequences transmitting inhibitory signals in intracellular regions [5]. LIRs contain multiple members such as LIR1, also named Ig-like transcript 2 (LIT2), LIR2/ILT4, etc. The ligands of LIR1/ILT2 are multiple MHC class I molecules (HLA-A, HLA-B, and HLA-G) [36–38] and UL18 glycoprotein from human cytomegalovirus [5]. The signaling pathways of LIRs and CD49/NKG2A are similar to IKIR inhibitory signaling pathways [8, 13], which can recruit SHP-1 to block the activating signals. The role of LIRs and CD94/NKG2A is to establish a threshold for NK cell activation to protect the normal cells. However, when the host is in a pathological state, the expression and function of those inhibitory receptors are abnormal, which would not be conducive to disease development. Scientists can research targeted drugs to restore the cytotoxicity of NK cells according to the mechanism of those inhibitory receptors.

### 3.3. ITIM-Containing Non-MHC Ligand Receptors

Besides the above receptors, NK cells still express many other intracellular ITIM-containing receptors on the surface [13, 39]: NKR-P1 (CD161), KLRG1, Siglec-7 (CD328), LAIR-1 (CD305), CEACAM-1, PILR*α*, TACTILE (CD96), TIGIT, etc. Among them, TIGIT, a non-MHC-I molecule-dependent inhibitory receptor, can recognize CD113, CD112, and CD155. And TIGIT is correlated to the maturation of NK cells and NK cell-mediated autoimmune tolerance [40].

## 4. Activating Receptors

There are multiple MHC-dependent or MHC-independent activating receptors on NK cells such as NKG2D, NCRs, and 2B4. Under physiological conditions, inhibitory receptors play a leading role in preventing NK cells from killing normal cells. However, when the MHC class I molecules on target cells are attenuated or absent, or the specific ligands directly recognize activating receptors, the inhibitory signal is weakened and the activation signal is enhanced, resulting in NK cells exhibiting killing effects. Activating receptors cannot activate NK cells on their own, except for CD16. Therefore, the activation of NK cells requires the synergy of multiple receptors.

### 4.1. Natural Cytotoxic Receptor (NCR)

NCRs are specific surface markers of NK cells, as well as major activating receptors of NK cells. The NCR family includes three members: NKp46 (NCR1, CD335), NKp44 (NCR2, CD336), and NKp30 (NCR3, CD337) [6].

NKp46-encoding genes are on human chromosome 19. The intracellular region of NKp46 does not have ITAM sequences. However, the transmembrane region of NKp46 contains an arginine residue that takes charge of associating with adaptors, Fc*ε*RI*γ* and CD3*ζ*, which can conduct activating signals [41]. The ligands of NKp46 include [42] (1) tumor cell ligands, such as ligands from melanoma and myeloma (but most of the tumor cell ligands are still unknown); (2) viral ligands, such as hemagglutinin (HA), hemagglutinin neuraminidase (HN); (3) bacterial ligands, such as vimentin on the mycobacterium tuberculosis-infected cell surfaces; and (4) parasitic ligands, such as Plasmodium falciparum erythrocyte membrane protein (PfEMP1). In addition, NKp46 can also identify complement factor P and unknown ligands on the hepatic stellate cells and pancreatic B cells, inhibit liver fibrosis, and participate in the pathogenesis of type I diabetes. All of the mature NK cells express NKp46 which acts a crucial role in triggering NK cell killing. The expression of NKp46 is related to the cytotoxic effect of NK cells [43]. The binding of NKp46 with the ligand can promote the killing ability of NK cells, increase the secretion of IFN-*γ* and TNF-*α*, and participate in the process of anti-infective immunity and killing tumor cells.

**Figure 2:**
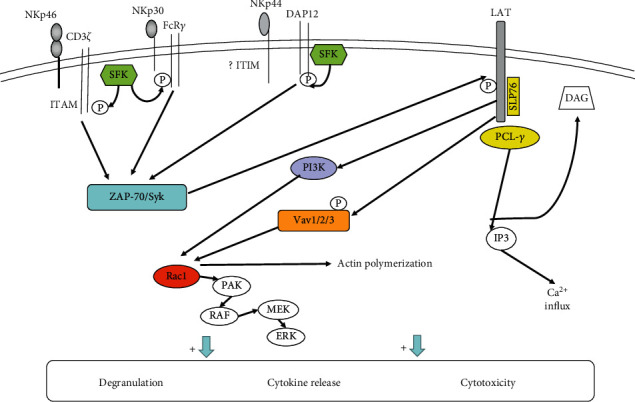
Model of signaling pathways by NCRs. After binding to their ligand, NKp46 and NKp30 associate with the adaptors Fc*ε*RI*γ* and/or CD3*ζ*, while NKp44 binds to DAP12. Furthermore, ITAMs in the adaptor protein intracellular regions are phosphorylated by SFK. Phosphorylated ITAMs activate the tyrosine kinases Syk and/or ZAP70. This recruits and activates downstream molecules such as PLC*γ*, PI3K, and Vav1/2/3 with the help of LAT. PLC*γ* further causes Ca^2+^ influx. PI3K and Vav1 can recruit the small G protein Rac1 and activate the MAPK signaling pathway through cascade phosphorylation of the PAK1–MEK–Erk signaling pathway. In addition, the intracellular region of NKp44 contains an ITIM sequence that may be related to inhibitory signaling, but the specific mechanism is not yet clear.

NKp46 transmits activating signals through ITAM-related receptors, which is similar to how most activating signals of NK cells are transmitted. The ITAM-containing adaptor proteins mainly include DAP12, FcR*γ*, DAP10, and CD3*ζ*. NK cells can constitutively express the type I transmembrane proteins Fc*ε*RI*γ*, CD3*ζ*, and DAP12. After binding to the ligands, NKp46 associates with the adaptor proteins CD3*ζ* and Fc*ε*RI*γ* [42]. Then, the ITAMs of adaptors are phosphorylated, which may be mediated by Src-family kinases such as Lck and Fyn [44]. Through the SH2 domain, the phosphorylated ITAM recruits and activates tyrosine kinases such as Syk and/or ZAP70 [45, 46]. The tyrosine kinases activate transmembrane adaptor proteins such as LAT and NTAT, leading to the activation of downstream molecules such as phospholipase C (PLC*γ*), phosphatidylinositol-3-OH kinase (PI3K), and Vav1, Vav2, and Vav3. PLC*γ* further causes Ca2^+^ influx; PI3K and Vav1 recruit the small G protein Rac1 and induce cascade phosphorylation through the PAK1-MEK-Erk signaling pathway, further activating the MAPK signaling pathway and other reactions [9, 47–51]. Ultimately, these signaling cascades promote actin cytoskeleton rearrangement, degranulation, cytotoxicity, and the gene expression of cytokine or chemokine (Figure 2). NKp46 acts as a coactivation receptor which triggers NK cell cytotoxicity by synergistic effects with other activating receptors. Researches confirmed that NKp46 could coengage with 2B4, CD2, NKG2D, and DNAM-1, transmitting activation signals to enhance the Ca^2+^ flux of NK cells further [52, 53]. However, we still do not know if synergistic signals of NKp46 and other coactivation receptors are the same as the synergistic signals of NKG2D and 2B4.

The coding genes of NKp30 and NKp44 are both on human chromosome 6. The ligands of NKp30 contain the following [42]: (1) tumor cell ligands, such as B7-H6, BAG6/BAT3 (BCL2-associated athanogene 6/nuclear HLA-B-associated transcript-3 protein), and galectin-3; (2) viral ligands, such as HA of vaccinia virus and poxvirus and pp65 of human cytomegalovirus; and (3) parasitic ligands, such as Plasmodium falciparum erythrocyte membrane protein (PfEMP1). In addition, all NCRs, including NKp30, can recognize heparan sulfate glycosaminoglycans (HS-GAGs), which are significantly upregulated in tumor cells. The expression and signal transduction of NKp30 are similar to those of NKp46. NKp30 makes synergistic reaction with NKp46 and NKp44 in triggering the cytotoxicity of NK cells. Delahaye et al.'s group transfected NK cell lines with NKp30a, NKp20b, and NKp30c separately and found that NKp30a and NKp30b were immunostimulating subtypes that could mediate the production of Th1 cytokines, while NKp30c enhanced the secretion of IL-10 and transmitted suppressive signals through the rapid phosphorylation of p38 MAPK [54]. Overall, NKp30 plays an important role in anti-infection immunity and antitumor immunity and is involved in tumor immune escape mechanisms. However, how it works still needs to be explored. Studies have found that soluble BAG6, a specific ligand of NKp30, could be detected in chronic lymphocytic leukemia (CLL), and the plasma levels of soluble BAG6 were higher at the advanced disease stages [55]. Then, they found that the soluble BAG6 released from CLL cells could inhibit the cytotoxicity of NK cells [55]. In contrast, exosomal BAG6 could enhance the killing function of NK cells [55]. As we all know that tumor microenvironment can influence the antitumor effect of NK cells, so the phenomenon that one molecule plays opposite acts on increasing the complexity of antitumor immunity. This may provide a new idea for increasing the NK cell killing effects as immunotherapeutic strategies.

NKp44 is only expressed on activated NK cell surfaces as a specific marker of activated NK cells. NKp44 ligands include the following [42]: (1) tumor cell ligands, such as proliferating cell nuclear antigen (PCNA), platelet-derived growth factor DD (PDGF-DD), nidogen-1, and NKp44L (NKp44L, an isomer of mixed-lineage leukemia-5 protein (MLL5), is expressed in tumor cells and transformed cells that can improve cell sensitivity to the cytotoxicity of NK cells [56]); (2) viral ligands, such as HA and HN; and (3) bacterial ligands, such as Mycobacterium tuberculosis cell wall components. Furthermore, some subtypes of HLA-DP are also ligands of NKp44 [57]. The transmembrane region of NKp44 contains Lys residues that can associate with KAPAP/DAP12 and transmit activation signals through the ITAM of KAPAP/DAP12. DNAX-activation protein of 12 kDa (DAP12; also named as killer cell-activating receptor-associated protein (KARAP)) is an adaptor protein containing a single ITAM in its intracellular domain [9, 10, 58]. The tyrosine residues in ITAM domain are rapidly phosphorylated under the action of the tyrosine kinase Syk after NKp44 receives stimulation. Further phosphorylation of the ITAM recruits Syk and ZAP-70. This pathway mediates downstream signal transduction and then activates NK cell function [9, 45] (Figure 2). Research has found that the intracellular domain of NKp44 contains a sequence consistent with the ITIM sequence [59]. Further studies have shown that this special sequence of NKp44 could be efficiently phosphorylated, but it was not able to inhibit NK cell function by recruiting of SHP-1, SHP-2, and SHIP [59]. Next, it still needs more researches to explore the mechanism of ITIM sequence as well as the phosphorylation of NKp44 and its function in signal conduction. Another study found that tumor cells could overexpress PCNA, which can associate with HLA I molecules forming an inhibitory ligand complex. NKp44 can recognize the complex ligand and suppress the NK cell killing activity through its ITIM sequences [60, 61].

The expression of NCRs can be influenced by many factors, such as cytokines, drugs, disease status, and epigenetic changes. For example, IL-2 increases the expression of NKp46 as well as enhances the killing effect of NK cells [62]. On the other hand, transforming growth factor-*β* (TGF-*β*) can downregulate the transcriptional level of NKp30 [63]. Drugs can also affect NCR expression. For instance, prolactin can upregulate the expression of NKp30 and NKp46, whereas corticosteroids have the opposite effect [64]. Meanwhile, the expression level of NCR on NK cells and the expression level of NCR ligands on tumor cells were related to the cytotoxicity of NK cells [42]. Furthermore, studies have found that multiple genes associated with NK cell surface receptors are upregulated following epigenetic changes [65]. TCR*β*^−^NKp46^+^ cells increase in the spleen, liver, and bone marrow of Ezh2^−/−^ mice, and human NK cells that expressed NKp46 also increase when selectively inhibiting Ezh2 activity, in vitro [66]. Further investigations on the influence factors of NK cell-activating receptors will be beneficial for understanding the roles of NK cells in various diseases and provide more ideas for NK cell-based immunotherapies in cancer.

### 4.2. NKG2D

NKG2D is a member of CL-SF and effects in activation signal transduction. It is a major activating receptor expressed on NK cells and CD8^+^ T cells in a dimer form [67]. The coding genes of NKG2D are located on chromosome 12 [67]. NKG2D can recognize multiple ligands such as MHC class I chain-related molecules (MICA and MICB) and human cytomegalovirus ULl6-binding proteins (ULBP1, ULBP2, ULBP3, ULBP4, ULBP5, and ULBP6) [67–70]. There are two different adaptor proteins, DAP10 and DAP12, both of which can associate with NKG2D and mediate activation via two different signaling pathways [71]. Studies have shown that there are two NKG2D splicing variants in mouse NK cells [72]. Resting NK cells express the longer protein (NKG2DL), which can associate with DAP10 only [73]. In contrast, activated NK cells express the shorter protein (NKG2DS) which can associate with either DAP10 or DAP12. Human NK cells express NKG2DL simply, so the intracellular tail of human NKG2D associates with DAP10 exclusively.

**Figure 3:**
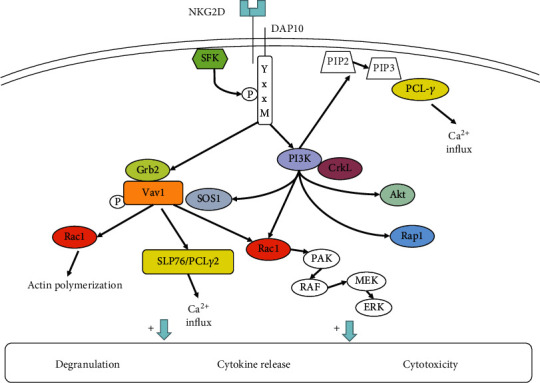
NKG2D signaling pathways. After DAP10 associates with NKG2D, it can bind to PI3K and the Grb2-Vav-1-SOS1 complex. The Grb2-Vav-1-SOS1 complex activates SLP76, PLC*γ*2, and Rac1. Rac1 can activate the MAPK signaling pathway and promote actin polymerization. SLP76/PCL*γ*2 causes Ca^2+^ influx. Furthermore, it leads to cellular degranulation and secretion of cytokines. PI3K can recruit PCL-*γ*, Rac1, and Akt signaling molecules to activate NK cell function. In addition, PI3K can recruit the SOS1-Grb2-Vav1 complex via SOS1 and recruit the small Ras-family GTPase Rap1 with the help of CrkL to achieve cytotoxicity.

As a critical activating receptor, through noncovalent binding to the adaptor protein DAP10, NKG2D can achieve multiple forms of signal transduction through phosphorylation by activating mitogen-activated protein kinase (MAPK) and Janus kinase (Jak)/signal transducer and activation of transcription (STAT) signaling [68, 74]. The intracellular segment of DAP10 contains a YxxM motif, which can bind to p85 PI3K, Grb2, and Shc [9]. Experimental results indicated that after the cross-linking of DAP10 and NKG2D, DAP10 could bind p85 PI3K and the Grb2-Vav-1-SOS1 complex to activate Akt/PKB [75–77]. However, there is no evidence that DAP10 can recruit Syk and/or ZAP-70 [75]. This indicated that DAP10 might transmit activation signals through different pathways than DAP12 and Fc*ε*RI*γ*. After DAP10 recruits PI3K and the Grb2-Vav1 complex, SLP76 and PLC*γ*2 are activated [51, 75, 77]. Activation signals eventually promote Ca^2+^ influx, cellular degranulation, and secretion of cytokines. Giurisato et al. reported that PI3K catalyzed the generation of PIP3, which can recruit the SOS1-Grb2-Vav1 complex via SOS1 [78]. Segovis et al. found that the interaction between p85 PI3K and the adaptor protein CrkL was required for NK cell activation [79]. PI3K and CrkL can further recruit the small Ras-family GTPase Rap1, which is necessary for NKG2D-mediated cytotoxicity, conjugate formation, and MTOC polarization [79] (Figure 3).

The expression and function of NKG2D are modulated by multiple factors. The ligands of NKG2D can affect the function of NKG2D. MICA can upregulate the expression of NKG2D and downregulate the expression of the inhibitory receptors NKG2A, NKG2B, and KIR2DL1. And then, NKG2D stimulates the cytotoxicity of NK cells on tumor cells. In contrast, soluble MICA can suppress the expression of NKG2D and inhibitory receptors [80]. Similarly, soluble ULBP also downregulate NKG2D expression on NK cells [80]. The soluble NKG2D ligands (NKG2DL) released from tumor cells may be a means of tumor immune escape in tumor microenvironment. In contrast, the binding of NKG2D to NKG2DL on the tumor cells can promote the killing effect of NK cells on tumor cells. However, tumor cells can change the expression of NKG2DL with various mechanisms to escape the attack mediated by NKG2D [81]. Martinet et al. showed that tumor cells secreted PGE2, inhibiting the activating signals of NKG2D, NCR, and CD16 on NK cells, thereby inhibiting tumor cells from being attacked by NK cells [82]. The mechanism may be related to the activation of EP2/EP4 receptors on NK cells via PGE2, which activates type I PKA and leads to Csk phosphorylation. Csk mediates Lck inactivation, preventing it from allowing activating receptors to bind to adaptor proteins, and then inhibits the transmission of activating signals. Sustained activation of NKG2D on NK cells decreases the reaction of other receptors, such as CD16 and NKp46 [83]. Otherwise, when NKG2D is combined with the natural receptor MICB, it will cause rapid endocytosis and degradation of NKG2D and DAP10 [84]. Studies have found that epigenetic changes regulate the expression and function of NKG2D [66]. Researchers found that higher levels of NKG2D on NK cells compared to wild type could be detected during Ezh2 deletion or inhibition of Ezh2 activity [66]. Researchers established an NKG2D-deficient mouse model (Klrk1^−/−^) and found that NKG2D deficiency affected maturation of NK subsets, leading to decreased NK cell numbers [85]. Using the Klrk1^−/−^ mouse model, researchers have found that Ezh2 activity inhibition can enhance the NK cell development, which requires the NKG2D expression [66]. Furthermore, inhibition of Ezh2 activity can upregulate the NKG2D-dependent cytotoxicity [66].

NKG2D activates NK cells by cooperating with other activating receptors, such as CD16, NKp46, and 2B4 [53, 86]. The synergistic activation signals will be introduced below. It is noteworthy that activation of NK cells is tightly associated with the signals of coactivation receptors. NKG2D also participates in regulating the function of other receptors. In 2018, Jelencic et al. found that Klrk1-/- mice had a stronger ability to inhibit tumor and cytomegalovirus infection during NK cell development than wild-type mice [87]. Subsequently, they found that deficiency of NKG2D or DAP12 could downregulate CD3*γ* and ZAP70, resulting in the upregulation of NCR1 signals [87]. This indicated that NKG2D can regulate the expression of NCR1 by setting an activation threshold.

### 4.3. CD244 (2B4)

**Figure 4:**
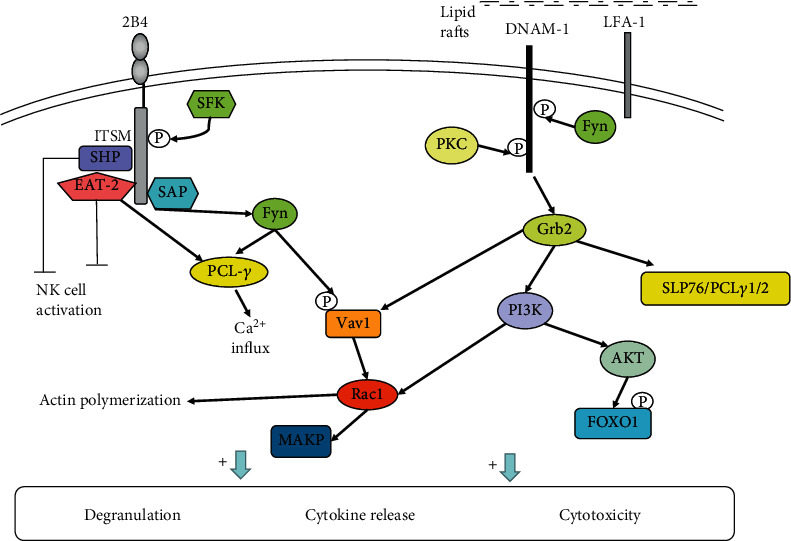
Signaling pathways of 2B4 and DNAM-1. Binding of 2B4 to CD48 promotes ITSM phosphorylation and recruits the adaptor SAP. SAP combines with ITSM to recruit Fyn, and Fyn phosphorylates the downstream proteins PLC*γ* and Vav1 to activate NK cells. 2B4 can also recruit the phosphatases SHP-1/SHP-2 in the absence of SAP, transmitting inhibitory signals. The 2B4-EAT2 complex may have opposite functions in NK cells. DNAM-1 binds to its ligand, and it is phosphorylated by PKC and Fyn, which promotes the interaction between DNAM-1 and LFA-1. Eventually, DNAM-1 is recruited to the lipid raft and binds to the adaptor Grb2. Binding of DNAM-1 to Grb2 enables PI3K, Vav1, SLP76, and PLC*γ*1/2 to be recruited and then activates the AKT and ERK signaling pathways, thus triggering degranulation and calcium mobilization. In addition, activated AKT catalyzes FOXO1 phosphorylation. Phosphorylated FOXO1 is transferred from the nucleus to the intracellular space, where it is inactivated and degraded, regulating NK cell cytotoxicity and exerting antitumor effects.

2B4 belongs to the signaling lymphocytic activation molecule (SLAM) family of CD2-related receptors [88]. It is expressed on all NK cells, CD8^+^ T cells, monocytes, and other immune cells [89]. 2B4 takes part in NK cell activation and participates in leukocyte differentiation [89, 90]. The specific ligand of 2B4 is CD48, which is also a member of CD2 subfamily. CD48 is generally expressed on hematopoietic-origin cells and parts of EBV-infected B cells. The intracellular region of 2B4 contains immunoreceptor tyrosine-based switch motifs (ITSMs), which are composed of TxYxxV/1 [91]. The SH-2-containing adaptor proteins SAP, EAT-2 (human), and ERT (rat) can associate with ITSM sequences [91, 92]. The binding of 2B4 to ligands causes ITSMs to undergo tyrosine phosphorylation under the action of SFK and recruits adaptor proteins. In humans, the ligand is mainly SAP. SAP combines with ITSMs to recruit FynT [93, 94]. FynT phosphorylates downstream proteins such as PLC*γ* and Vav1 [95]. The 2B4-SAP complex can trigger NK cell activation. Otherwise, SAP is capable of promoting the combination of EAT2 and 2B4, which indicates that SAP plays a crucial role in EAT2-related signal pathways [96]. Some studies showed that EAT-1 suppressed the cytotoxicity and IFN-*γ* secretion of NK cells [97]. In contrast, some studies proved that EAT-2 may be capable of promoting NK cell cytotoxicity, granule polarization, and degranulation by the activation of PLC*γ*, Ca^2+^, and Erk [98, 99]. Overexpression of EAT-2 can enhance the NK cell antitumor activity [100]. The function of EAT-2 may be correlated to the environment of NK cells. When SAP is absent, 2B4 can conduct inhibitory signals through recruiting SHP-1, SHP-2, SHIP, and Csk and block NK cell activity [98, 101] (Figure 4). Research has proven that 2B4 can dephosphorylate P27 by activating the SHP-2 signaling pathway, which plays an important role in maintaining the development of leukemia-initiating stem cells [102]. However, there is much work to be done about how do SHP-1 and SHP-2 work in the 2B4 signal pathway. Does 2B4 conduct inhibitory signals through SHP-1or SHP-2? It is needed to mention that deletion or functional mutation of SAP can result in a severe hereditary immunodeficiency disease, X-linked lymphoproliferative disease (XLP). SAP function loss results in a decrease in NK cell anti-infection ability in XLP patients, and patients are having difficulty to control Epstein-Barr virus infection [103]. Current studies suggest that 2B4 exhibits different effects during NK cell development and maturation. In the early stage of NK cell maturation, 2B4 only inhibited NK cells and blocked the killing activity of NK cells, suggesting that 2B4 contains potential suppression effect [104]. After NK cells mature, the function of 2B4 depends on the balance between different signaling molecule complexes. Studies have shown that the function of 2B4 is influenced by several factors: the expression density of 2B4, the expression level of its ligands, and the relative content of certain adaptor molecules [105].

**Figure 5:**
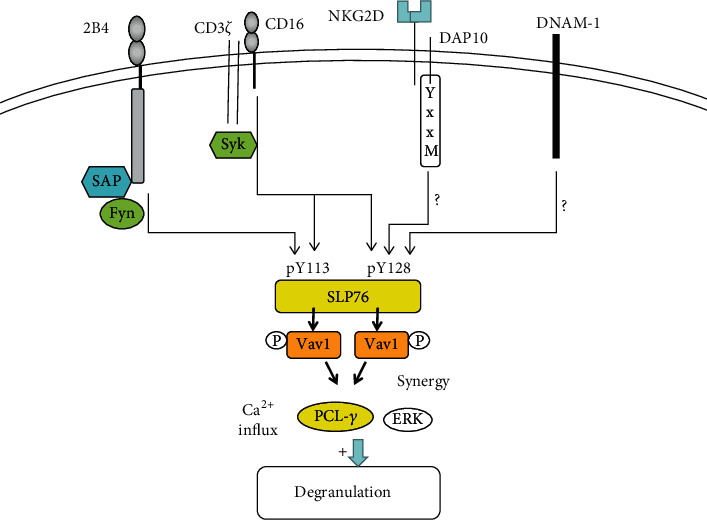
Synergistic activation of coactivation receptors is Vav1-dependent; NK cells promote the cytotoxicity and the secretion of INF-*γ* by the synergistic effects of NKG2D and 2B4 and 2B4 and DNAM-1. After coactivation receptors engaged, synergistic signals are integrated at SLP76. The phosphorylation of SLP76 induced by 2B4 was Fyn-dependent, whereas the phosphorylation of SLP76 in Y128 induced by NKG2D or DNAM-1 was SYK-independent. In contrast, CD16 can phosphorylate both Y113 and Y128 of SLP76 and sufficient to induce NK cell action on itself. Phosphorylated SLP76 associates with Vav1, resulting in phosphorylation of PLC*γ*2 and ERK, which promotes degranulation and INF-*γ* secretion of NK cells.

Similar to NKG2D, 2B4 acts as an NK cell coreceptor in combination with its ligand CD48 to take effect [106]. Its function of transmitting signals in most cases relies on the participation of other activating receptors, such as NCRs, NKG2D, and CD226. Then, 2B4 and the coactivation receptor activate NK cell cytotoxic effect and IFN-*γ* production synergistically and play an important part in antiviral and antitumor immunity. But how does synergistic signal transmit? Researchers found that coactivation receptors, NKG2D and 2B4 or 2B4 and DNAM-1, could conduct synergistic signals by phosphorylating Vav1 and then, PLC*γ*2 and ERK were phosphorylated [33]. Further, phosphorylated PCL*γ*2 and ERK induced the Ca^2+^ mobilization, cytotoxic degranulation, and the secretion of INF-*γ* [33]. NKG2D and 2B4 each one activated alone could phosphorylate Vav1, but only NKG2D and 2B4 synergy could induce degranulation [33], which may be associated with the ubiquitination in Vav1 induced by c-Cbl. c-Cbl could inhibit the activation of NK cells through a Vav1-dependent way, and consequently, synergy of coactivation receptors is required to overcome negative regulation of c-Cbl [33]. On the other hand, the activation signals from different coactivation receptors need to be integrated before Vav1, which need the phosphorylation of adaptor protein SLP76 by each coactivation receptor [107] (Figure 5). The phosphorylation of SLP76 in Y113 could be induced by the cross-linking of 2B4, and the phosphorylation of SLP76 in Y128 could be induced by the cross-linking of NKG2D or DNAM-1 [107]. For Y113 or Y128, each tyrosine phosphorylation of SLP76 was required for the synergistic activation of NK cells [107]. Therefore, the cooperation of NKG2D, 2B4, and DNAM-1 is necessary for NK cell activation. But how coactivation receptors phosphorylate the SLP76 still needs to be researched. Researchers found that the phosphorylation of SLP76 induced by 2B4 was Fyn-dependent, whereas the phosphorylation of SLP76 in Y128 induced by NKG2D was SYK-independent. Thus, there is much work required to be done about the signals for synergistic activation of NK cells, in the future.

NK-T-B antigen (NTB-A) belongs to the SLAM family and can be detected on all NK cells, T cells, and B cells [88]. The action mechanism of NTB-A is analogous to 2B4 [108]. It also plays a synergic effect with activating receptors to assist in the NK cell activation. Studies have shown that 2B4 and NTB-A can straightly recognize the HA of influenza virus via sialylation and induce the killing function of NK cells through costimulation. The virus can counteract this process by neuraminidase (NA), which indicates the action of 2B4 and NTB-A in antiviral immunity [109].

### 4.4. DNAM-1 (CD226)

The DNAX-activating molecule (DNAM-1) gene is located on chromosome 18. DNAM-1 belongs to the Ig-SF and is expressed on the surfaces of NK cells, T cells, and monocytes, which participated in the formation of immunological synapses. It can transmit activating signals through synergetic effects with lymphocyte function-associated antigen 1 (LFA-1) or 2B4. The ligands of DNAM-1 are CD112 (nectin-2, PRR2) and CD155 (PVR, Necl4), which are expressed in some immune cells such as monocytes, DCs, activated CD4^+^ T cells, and tumor tissue [110]. In DNAM-1-mediated cytotoxicity, PVR is the main ligand. In addition to DNAM-1, PVR can also interact with CD96 (TACTILE) and TIGIT. Under normal conditions, autologous cells express low levels of PVR, and TIGIT combined with PVR inhibits NK cell activation. However, during tumor formation, malignant cells highly express PVR, which can bind to DNAM-1 and CD96 activating the antitumor effects of NK cells [39, 111, 112].

The intracellular region of DNAM-1 contains a special signaling structure, which has four tyrosine residues Y293, Y300, Y322 (equivalent to Y319 in the murine orthologue), and Y325 and one serine residue, S329 (equivalent to Y326 in the murine orthologue) [113]. In a mouse model, the phosphorylation of Y319 and S326 has been shown to play a key role in DNAM-1 signaling pathways. DNAM-1 associates with LFA-1, which induces tyrosine kinase Fyn to phosphorylate Y319 on CD226. LFA-1 and DNAM-1 can physically bind during the process of immune synapse formation [113]. When LFA-1 is deficient, NK cells lose the killing function mediated by CD226 [113]. The phosphorylation of Y319 on DNAM-1 is very important for the function of LFA-1, and LFA-1 can promote the tyrosine kinase Fyn to phosphorylate Y319 on DNAM-1 in turn [114]. When the immune synapse is established between NK cells and target cells, LFA-1 binds to ICAM-1 in the target cells. At the same time, DNAM-1 of NK cells binds to its ligand, and S326 in the intracellular region of DNAM-1 is phosphorylated by PKC, which promotes LAF-1 to interact with DNAM-1 via Y319 phosphorylated by tyrosine kinase Fyn in the intracellular region of DNAM-1 [113, 114]. Eventually, DNAM-1 is recruited to the lipid raft as well as aggregates at the immune synapse site where a large number of signals occur. Furthermore, DNAM-1 binds to adaptor Grb2 through phosphorylated Y319 cooperating with N321. Binding of DNAM-1 to Grb2 enables PI3K, Vav1, SLP76, and PLC*γ*1/2 to be recruited and then activates the AKT and ERK signaling pathways, triggering degranulation and Ca^2+^ mobilization [39, 107, 114, 115]. Consequently, NK cell function is activated (Figure 4). In addition, the intracellular region of the DNAM-1 molecule also contains motifs for binding to the isoforms of actin-binding protein 4.1G. 4.1G is an important molecule in the membrane cytoskeleton structure, which can interact with the membrane-associated guanylate kinase (MAGUK) homologue, and plays a part in the formation and anchoring of membrane protein complexes [116]. MAGUK molecules provide multiple functional domains to cluster multiple molecules related to activation such as membrane protein receptors, adhesion molecules, and intracellular signaling proteins at synapses, cell junctions, and polarized membrane functional regions [116]. The binding of these two protein families to the DNAM-1 molecule may be related to the formation of the cytoskeleton, the clustering of CD226 molecule, the narrowing of CD226 and LAF-1 molecules, and the entry of CD226 into lipid rafts [116].

DNAM-1 is a costimulatory receptor that plays an important role in antitumor and antivirus immunity. A study has shown that DNAM-1 can phosphorylate FOXO1 by activating AKT. Thus, FOXO1 is transferred from the nucleus to the intracellular space, where it will be inactivated and degraded, regulating NK cytotoxicity and playing an antitumor role [117]. In addition, one issue that deserves attention is that the functional status of DNAM-1 is strongly related to the expression extent of its ligand and inhibitory receptors, CD96 (TACTILE), TIGIT, and PVRIG [39]. The interaction and balance between these receptors are complicated, and we will not introduce it here.

### 4.5. Activating Killer Cell Immunoglobulin Receptors (AKIRs) and CD94/NKG2C

AKIRs are subtypes of KIRs with a shorter intracellular tail, and they conduct activation signals to activate NK cells. AKIRs can be divided into KIR2DS and KIR3DS the same as IKIRs. Their intracellular region does not contain ITIMs, but the transmembrane region can be noncovalently bound to adaptor proteins containing ITAM sequences [4]. CD94/NKG2C does not contain ITIMs in the intracellular region, but it has the ability of transmitting activation signals through noncovalently binding to adaptor proteins. KIR2DS, KIR3DS, and CD94/NKG2C are MHC-dependent activating receptors. KIR2DS1, KIR2DS2, and KIR2DS4 can recognize HLA-C. KIR3DS1 can recognize HLA-B. The ligand of CD94/NKG2C is HLA-E [30]. The adaptor protein of KIR2DS, KIR3DS, and CD94/NKG2C is DAP12, and their phosphorylation and downstream signaling pathways are the same as those described for NCRs [9, 10].

In addition, the activating receptors associated with NK cell function include CD16 (FcRII) recognizing antigen-antibody complexes, Tim-3 binding to galectin-9, NKp80 binding to activation-induced C-type lectin (AICL), CD28 recognizing CD80 (B7-1) and CD86 (B7-2), and CD2 binding to CD48 and CD58 (LFA-3) [8].

## 5. Conclusion

Activation and inhibition of NK cell function require the interactions between receptors and their corresponding ligands and are also closely related to the interactions between receptors. Understanding the mechanisms of function of NK cell receptor and its mediated signaling pathway is helpful in exploring the mysteries of NK cell function. Knowing more about how NK cells function regulation can help scientists to discover more about the role of NK cells in the pathogenesis of lots of immune diseases, as well as provide more strategies in NK cell immunotherapies. Although MHC-restricted inhibitory receptors need more extensive researches in regulating NK cell cytotoxicity and cytokine secretion, some targeted drugs have been explored for the effectiveness in solid tumors and hematological tumors, such as anti-KIR and anti-NKG2A mAbs [118]. NK cell-based immunotherapies are feasible because of the antigen-unrestricted cytotoxicity of NK cells on tumor cells. However, the expression of NK cell-activating receptors and specific ligands has individual difference in different patients. Thus, it is necessary to distinguish the individual difference and find out the inherent law of NK cell receptors in different diseases, in the future. Studying on the mechanism of NK cells in anti-infection immunity and antitumor immunity can provide more theoretical basis for exploring new targets of tumor and virus therapy. NK cells as innate immune cells also play parts in autoimmune reaction. Thereby, changes in the NK cell function and quantity participate in the pathogenesis of multiple autoimmune diseases. Our group found that NK cells had high expression of activating receptors NKp46 and NKG2D, low-expression of inhibitory receptor NKG2A, and increased cytotoxicity in patients with severe aplastic anemia (SAA) [119]. Thus, we speculated that NK cells suppressed the function of CD8^+^ T cells and played immunoregulation roles in the pathogenesis of SAA. Next, we want to start from the signal pathway, gene, epigenetic, and other aspects to explore the specific mechanism of NK cell receptors and function changes in SAA. In the future, we should pay more attention to the relationship between NK cells and different diseases, as well as how do those NK cell inhibitory receptors and activating receptors change and act. Ultimate goals are to provide new treatment breakthroughs applied in clinic by the researches of mechanism.
